# A SIV molecular clone that targets the CNS and induces neuroAIDS in rhesus macaques

**DOI:** 10.1371/journal.ppat.1006538

**Published:** 2017-08-07

**Authors:** Kenta Matsuda, Nadeene E. Riddick, Cheri A. Lee, Sarah B. Puryear, Fan Wu, Bernard A. P. Lafont, Sonya Whitted, Vanessa M. Hirsch

**Affiliations:** 1 Laboratory of Molecular Microbiology, NIAID, NIH, Bethesda, MD, United States of America; 2 Viral Immunology Section, OD, NIAID, NIH, Bethesda, MD, United States of America; University of Wisconsin, UNITED STATES

## Abstract

Despite effective control of plasma viremia with the use of combination antiretroviral therapies (cART), minor cognitive and motor disorders (MCMD) persist as a significant clinical problem in HIV-infected patients. Non-human primate models are therefore required to study mechanisms of disease progression in the central nervous system (CNS). We isolated a strain of simian immunodeficiency virus (SIV), SIVsm804E, which induces neuroAIDS in a high proportion of rhesus macaques and identified enhanced antagonism of the host innate factor BST-2 as an important factor in the macrophage tropism and initial neuro-invasion of this isolate. In the present study, we further developed this model by deriving a molecular clone SIVsm804E-CL757 (CL757). This clone induced neurological disorders in high frequencies but without rapid disease progression and thus is more reflective of the tempo of neuroAIDS in HIV-infection. NeuroAIDS was also induced in macaques co-inoculated with CL757 and the parental AIDS-inducing, but non-neurovirulent SIVsmE543-3 (E543-3). Molecular analysis of macaques infected with CL757 revealed compartmentalization of virus populations between the CNS and the periphery. CL757 exclusively targeted the CNS whereas E543-3 was restricted to the periphery consistent with a role for viral determinants in the mechanisms of neuroinvasion. CL757 would be a useful model to investigate disease progression in the CNS and as a model to study virus reservoirs in the CNS.

## Author summary

Despite effective control of plasma viremia with the use of combination antiretroviral therapies, neurologic disease resulting from HIV-infection of the central nervous system (CNS) persists as a significant clinical problem. Non-human primate models are therefore required to study mechanisms of disease progression in the CNS. We generated an infectious molecular clone (CL757) of an SIV isolate from the brain of a macaque with neuroAIDS. This cloned virus induced neurological disorders in 50% of rhesus macaques infected but without rapid disease progression often seen in other commonly used animal models. Molecular analysis of tissues from macaques infected with CL757 revealed that the variants isolated from the CNS and the periphery became genetically distinct from one another. When co-inoculated with an AIDS-inducing, non-neurovirulent clone (E543-3), CL757 targeted the CNS consistent with its neurovirulence. CL757 would be a useful model to investigate disease progression in the CNS and as a model to study virus reservoirs in the CNS.

## Introduction

Entry of HIV to the CNS occurs early in the course of infection and can progress to HIV-associated dementia (HAD) or HIV encephalitis (HIVE) [1]. HAD is a neurological syndrome that affects 20–30% of infected individuals in the later stages of HIV-infection and includes a range of cognitive and motor disorders, such as impaired short-term memory, reduced concentration and leg weakness. These symptoms are presented along with behavioral changes such as social withdrawal and in extreme cases, near vegetative and mute states. While the introduction of combinational anti-retroviral therapy (cART) has reduced the incidence of HAD to 10% in infected individuals, a milder form of HAD, minor cognitive motor disorder (MCMD), has become more common and affects 30% of the HIV infected population [2–5]. In this syndrome, loss of cognitive and motor functions is less severe, however, MCMD is associated with a worse prognosis for HIV infected individuals [6–8]. Clinical determination of HAD is associated with the presence of multinucleated giant cells (MNGCs) at the time of autopsy, due to productive infection of macrophages and microglial cells, a hallmark of HIVE [9–13].

Neuropathological evaluation of HAD/MCMD progression in humans is not feasible since CNS tissue sampling, other than cerebral spinal fluid (CSF) as a surrogate, is limited to end-stage disease. This creates the need for an animal model that will allow for the study of HIVE pathogenesis. Simian immunodeficiency virus (SIV) infected rhesus macaques are widely used as a model for AIDS pathogenesis. Infection of these animals with neurotropic SIV can result in SIV encephalitis (SIVE/neuroAIDS), with neuropathologic findings reminiscent of HIV encephalitis in humans, including the presence of multinucleated giant cells (MNGCs). Also, infection of macaques with SIV allows researchers access to samples of any part of the brain throughout all stages of disease progression under controlled conditions.

Currently, two nonhuman primate models tend to dominate studies evaluating the mechanism of pathogenesis of SIVE/neuroAIDS. These include the use of immunomodulation by depletion of CD8^+^ lymphocytes prior to or following inoculation with strains of SIV (SIVmac251/239) that otherwise are inconsistent in inducing SIVE. When CD8^+^ cells are depleted from rhesus macaques, disease progression is rapid, resulting in AIDS accompanied by a high incidence of SIVE within 3 to 6 months after infection [14–17]. The advantage of this model is that progression to neuroAIDS is rapid and highly reproducible. However, since the host immune system is modified, it may not directly reflect the pathogenesis of HAD/MCMD in HIV infected individuals. The second model is the co-inoculation of the uncloned, immunosuppressive virus SIVsmB670 and a neurovirulent viral clone SIVmac17E-Fr, into pig-tailed macaques [18–21]. As with the CD8^+^ lymphocyte depletion model, pig-tailed macaques co-inoculated with both strains also reproducibly show rapid disease progression with accompanying CNS disorders. As with the CD8-depletion model, immunomodulation is required to observe neuroAIDS, in this case, B670 induces immunosuppression to allow more efficient replication of 17E-Fr. Although co-inoculation with uncloned virus complicates the analysis of the relative importance of the components of the complex viral populations, these studies suggested that the neurovirulent clone, SIVmac17E-Fr targets the CNS. Another disadvantage of this model is that the prevalence of CNS disease in rhesus macaques is relatively low, requiring the use of pig-tailed macaques that are limited in number for large-scale experiments.

We therefore developed a model that achieves encephalitis in rhesus macaques at a high frequency without immunomodulation by repeated passage of viruses isolated from the brain of rhesus with SIVE [22]. Whereas the initial SIVsmE543-3 clone was only rarely associated with neuroAIDS and replicated inefficiently in monocyte derived macrophages (MDM), the final uncloned SIVsm804E isolate induced SIVE with relatively high frequency and replicated efficiently in MDM. Using construction of chimeric viruses of SIVsmE543-3 with portions of the env, nef and LTRs cloned from the neurovirulent SIVsm804E, we identified four amino acid substitutions in the cytoplasmic tail of gp41 that enhance replication in macrophages and were associated with enhanced BST-2 antagonism [23]. Rhesus macaques inoculated with the parental virus SIVsmE543-3 engineered to contain these four mutations exhibited higher cerebrospinal fluid (CSF) viral load than animals infected with wild type virus [23]. However, despite increased CSF viral load, this clone did not fully recapitulate the pathogenesis of the uncloned isolate [23], consistent with the hypothesis that other viral determinants were required for full neuropathogenesis. It thus was critical to develop clones fully representative of the neurovirulent strain.

In the present study, we generated a full length infectious clone from this neurovirulent isolate, SIVsm804E-CL757 (CL757). CL757 replicates efficiently *in vitro* in activated peripheral blood mononuclear cells (PBMCs) and monocyte-derived macrophages (MDMs), which has been reported to be a predictor of neurotropism [24–26]. Animals developed clinical symptoms of neurologic disease similar to what is seen in patients with HAD, such as loss of motor control and paralysis. Longitudinal analysis of cerebral spinal fluid (CSF) showed high viral titers in the CNS that correlated with the formation of brain lesions as seen by immunohistochemistry and SIV-specific *in situ* hybridization. This model also confirms studies that show compartmentalization of virus between the CNS and the periphery [27–32]. Sequences in the plasma and lymph nodes (periphery) showed more diversity of sequence than variants isolated from the brain and CSF (CNS) and showed divergent evolution from CL757 and convergence towards the parental strain SIVsmE543-3 (E543-3) [33], suggesting that CL757 is already highly adapted to the CNS environment. Co-inoculation of macaques with clone CL757 and the parental clone SIVsm-E543-3 demonstrated that CL757 specifically targets the CNS resulting in the development of neuroAIDS.

## Results

### Construction and characterization of the neurotropic clone CL757

**Fig 1:**
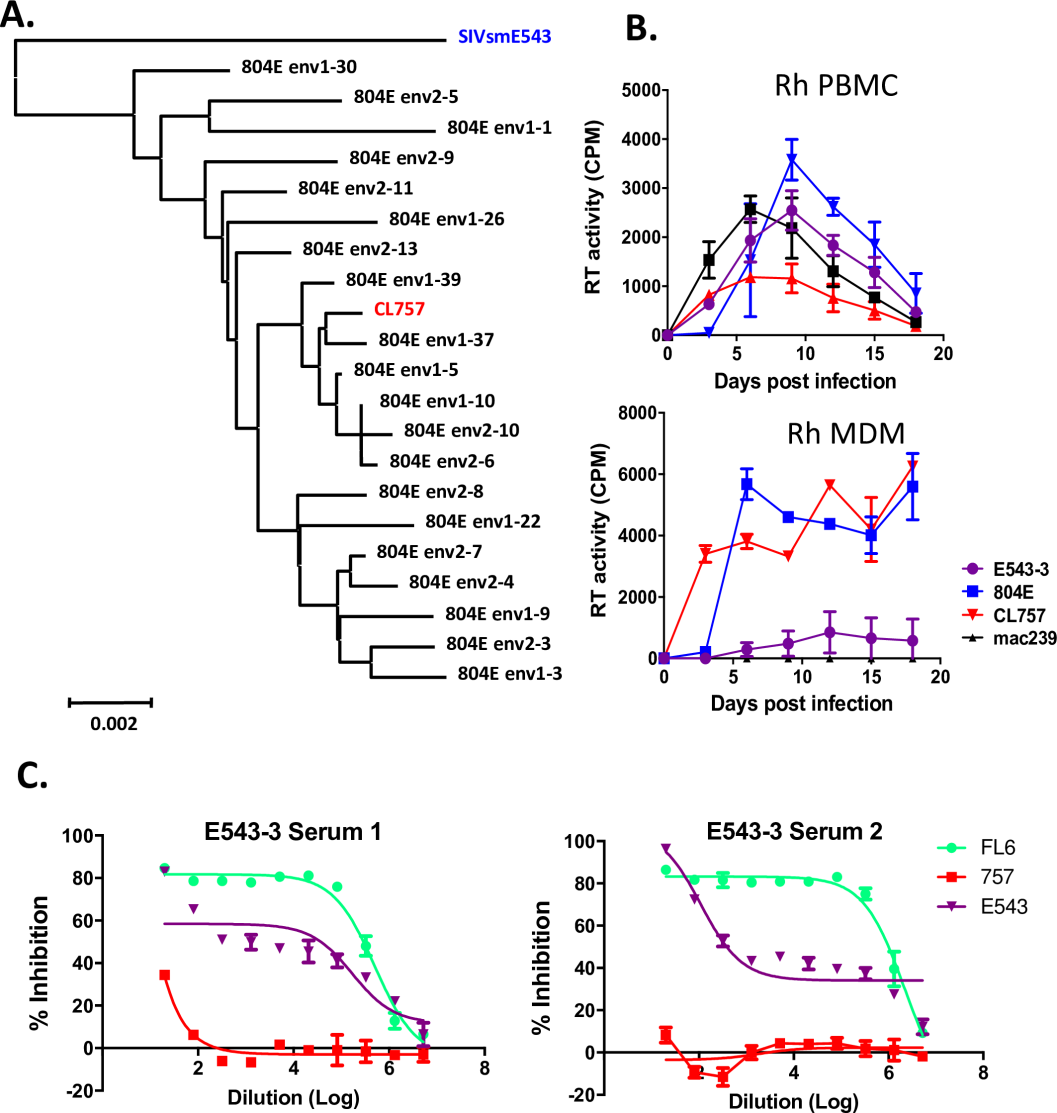
Biologic evaluation of full length CL757 derived from the uncloned neurotropic SIVsm804E. **A.** Phylogenetic analysis of envelope sequences amplified from uncloned SIVsm804E and CL757. Phylogenetic analysis of envelope nucleotide sequences was conducted on two independent stocks of uncloned SIVsm804E (indicated as env1 and env2), CL757 and SIVsmE543-3. **B.** Replication kinetics of CL757 in PBMCs and MDMs as compared to E543-3, Mac239 and 804E. Results, shown as cpm/ml of culture supernatant, are representative of three different SIV-naïve rhesus macaque donors tested in independent experiments (RhMO3, RhDCCW and RhDCRG). **C.** Percent inhibition of CL757, E543-3 and E660-FL6 replication as measured by RLU in the TZM-bl assay with serial dilutions of sera from E543-3-infected rhesus macaques were calculated.

In a previous study, we identified substitutions in the cytoplasmic tail of gp41 of viruses of a neurovirulent SIV isolate, SIVsm804E that were associated with enhanced antagonism of the host restriction factor, BST-2. These substitutions conferred enhanced replication in macrophages and higher cerebral spinal fluid viral load in infected rhesus macaques [23]. However, since these animals did not develop SIVE, this virus did not appear to recapitulate the full neurovirulence of the uncloned isolate, consistent with the contribution of other genetic determinants. Therefore, we generated a series of chimeric clones from the 804E isolate, mixing and matching between three different 2-LTR constructs and four clones of the entire viral genome spanning the structural, replication and regulatory genes (clones #1, 4, 5 and 7), resulting in 12 full-length SIV clones. As shown in Table 1, clones containing the viral genomes #1 and #4 in combination with any of the LTRs (CL414, CL444, CL616, CL646, CL717 and CL747) did not replicate in either PBMCs or MDMs and were not evaluated further. CL454 and CL484 efficiently replicated in MDMs with low levels of replication in PBMC, while CL656, CL686 and CL787 replicated in PBMCs only. CL757 was the only clone to replicate robustly in both PBMCs and MDMs. Since it has been previously reported that neurotropic viruses are able to replicate in both of these cell types *in vitro*, CL757 was chosen for further characterization. Phylogenetic analysis of the envelope sequence of CL757 revealed that it clustered with other envelope clones derived from the SIVsm804E isolate that was its source, indicating that it was generally representative of this isolate (Fig 1A).

**Table 1:** Infectivity and replication of clones from SIV804E Stock in Rhesus. PBMC and monocyte derived macrophages.

Clone Name	LTR	Genome	Rh PBMC	Rh MDM
414	4	804–1	-	-
444		804–4	-	-
454		804–5	+/-	+
484		804–8	+/-	+
4E4		E543	+	+
616	6	804–1	-	-
646		804–4	-	-
656		804–5	+	-
686		804–8	+	-
6E6		E543	+	+
717	7	804–1	-	-
747		804–4	-	-
757		804–5	+	+
787		804–8	+	-
7E7		E543	+	+

### In vitro characterization of CL757

We previously reported that the uncloned 804E, isolated after sequential *in vivo* passages of virus isolated from brains of infected macaques with SIVE, replicated more efficiently in MDMs compared to its parental strain E543-3 (E543-3) [22]. Therefore, we assessed the kinetics and extent of *in vitro* replication of CL757 in PBMCs and MDMs. Replication kinetics were compared to the T cell tropic virus SIVmac239 (Mac239), the original parent E543-3 and the uncloned 804E source of CL757. Under the same conditions, all viruses replicated in PBMCs, however, CL757 replication efficiency was less robust than Mac239, 804E and E543-3 (Fig 1B). In contrast, CL757 showed replication kinetics similar to that of the parental uncloned 804E in MDMs, contrasting with impaired replication of Mac239 and E543-3 viruses in MDMs (Fig 1B). Replication in MDMs is a characteristic feature of neurotropic SIVs and these results indicate that CL757 is a potential candidate as a neurotropic virus. We speculated that replication in PBMCs might also be essential for neurovirulence since the virus must first establish a systemic infection. Many macrophage-tropic SIVs have been reported to be very sensitive to neutralizing antibodies (NAb) [34, 35]. We used sera from rhesus macaques (n = 4) infected for over one year with the neutralization-resistant E543-3 virus to assess the sensitivity to Nab of CL757 as compared to E543-3 and a neutralization-sensitive clone of E660-FL6 [36]. Whereas FL6 was easily neutralized by these sera, NAb titers were significantly less robust for E543 and CL757 was not neutralized, consistent with a neutralization-resistant phonotype (Fig 1C).

### *In vivo* characterization of CL757

**Fig 2:**
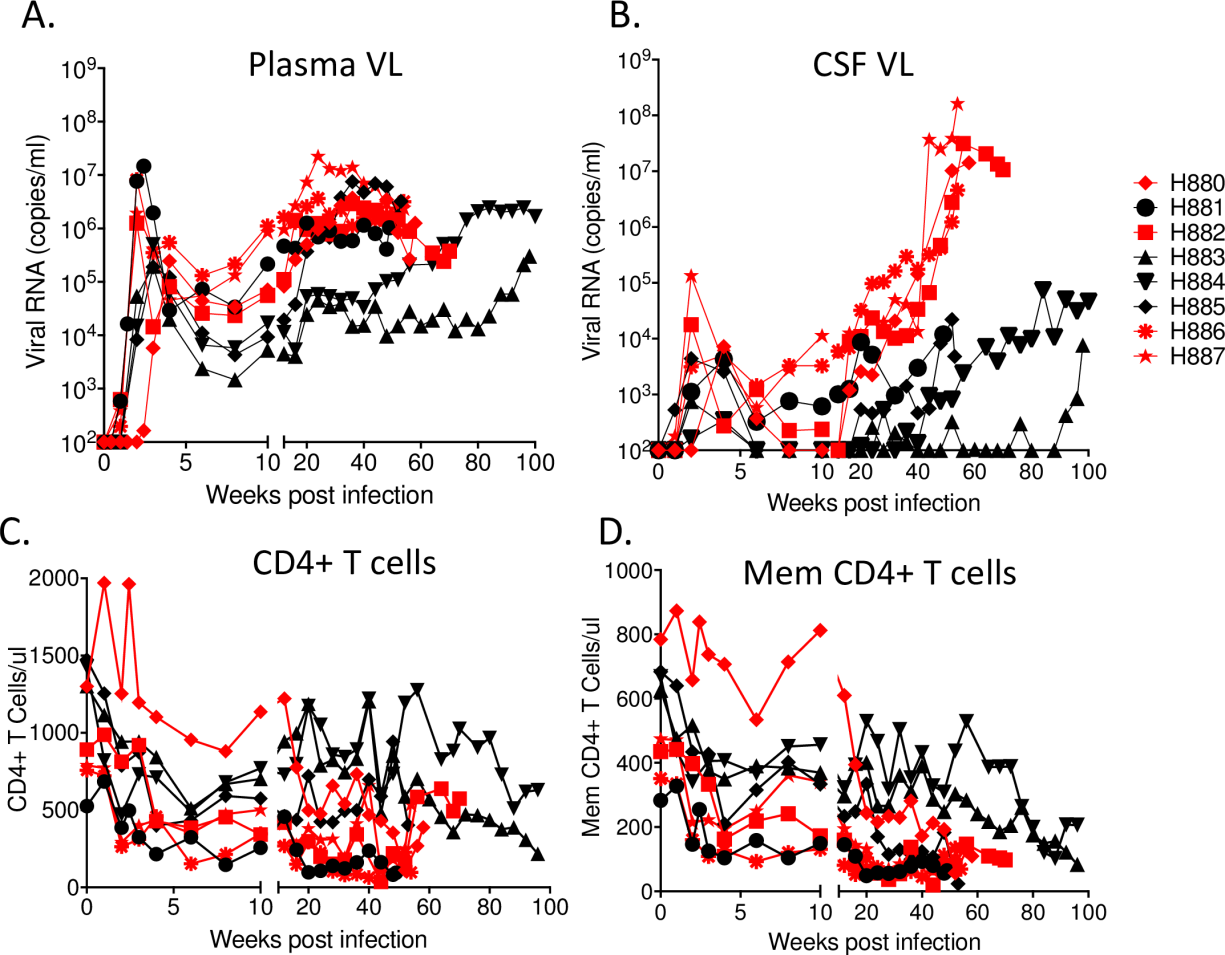
Replication of CL757 in rhesus macaques *in vivo*. (A) Plasma and (B) CSF viral RNA loads, and circulating (C) CD4+ T cells and (D) memory CD4+ T cells, of animals infected with CL757. Red symbols indicate animals that developed SIVE, and black symbols indicate animals that died with AIDS but without SIVE.

**Fig 3:**
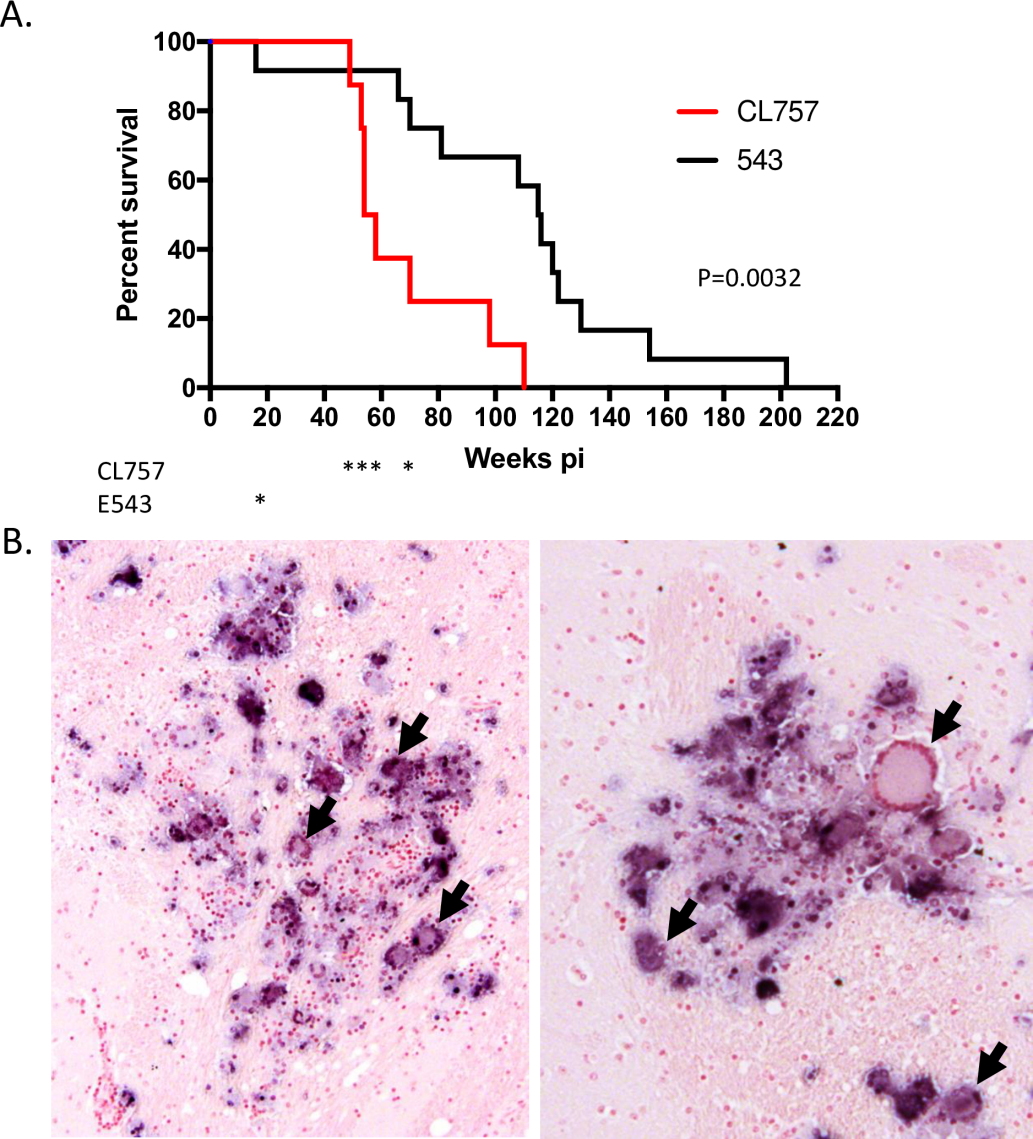
Clinical and pathologic endpoints in macaques inoculated with CL757. (A.) The cumulative survival of macaques infected with CL757 (Red) and E543-3 (Black) are shown as Kaplan Meier curves and compared by Log-Rank test (P = 0.0032). Asterisks below the graph indicate the death of animals with SIVE. B. SIV specific *in situ* hybridization of the brain parenchyma of a representative animal with SIVE (H880) showing characteristic glial nodules containing multinucleated giant cells (indicated by arrows). Dark blue pigment (NBT) indicate the presence of viral RNA associated with lesions.

To assess the *in vivo* replication capacity of CL757, eight naïve Indian origin rhesus macaques were intravenously inoculated with 500 TCID_50_ of CL757 (see Table 2). Robust viral replication was observed in the plasma of all infected animals with peak viral load ranging from 10^5^−10^7^ viral RNA copies/mL of plasma between two and three weeks post-infection (Fig 2A). In six of the eight animals infected (H880, H881, H882, H885, H886 and H887), plasma viral RNA initially declined by two to three logs but over time gradually increased to a set point range of 10^5^−10^7^ viral RNA copies/mL by week 20. The remaining two animals (H883 and H884) exhibited low peak viral loads, one to two logs lower as compared to the viral RNA set points of the other six study animals (2 x 10^4^ and 6 x 10^4^ viral RNA copies/mL of plasma, respectively). A gradual increase in viral RNA was observed after 84 weeks post-infection in H883 such that levels eventually achieved similar end-point levels as the others animals by the time of necropsy at 96 weeks post-infection (2 x 10^5^ copies/ml). Plasma viral load of H884 started to increase prior to H883 and reached to the same end-point level by 64 weeks post-infection (5 x 10^5^ copies/ml) and was maintained at this level until necropsy at 100 weeks post-infection. All study animals were eventually euthanized due to development of opportunistic infections or neuroAIDS (Fig 3A) and disease progression was significantly more rapid than in a historic cohort of macaques infected with the parental E543-3 cloned virus (log rank test, p = 0.0032). However, none of the animals infected with CL757 progressed rapidly to AIDS (i.e. in <6 months), with the first animal to be euthanized surviving until 49 weeks.

**Table 2:** Clinical, virologic and neurological outcome in macaques inoculated with CL757.

Animal ID*	Survival (weeks)	Plasma viral load (copies/ml)	CSF viral load (copies/ml)	Clinical symptoms of neurological disease	Inoculum	TRIM5 genotype
H880	58	1.3 x 10^6^	1.4 x 10^7^	Slow/staggered gait Arm tremors Hiding–hunched in back of cage	CL757	TFP/Q
H881	49	1.0 x 10^6^	1.2 x 10^4^	None	CL757	TFP/Q
H882	70	3.8 x 10^5^	1.0 x 10^7^	None	CL757	Q/Cyp
H883	98	3.0 x 10^5^	7.6 x 10^3^	None	CL757	Q/Cyp
H884	105	1.6 x 10^6^	4.6 x 10^4^	None	CL757	Q/Cyp
H885	53	3.2 x 10^6^	4.8 x 10^3^	None	CL757	Q/Q
H886	54	3.2 x 10^6^	4.6 x 10^6^	Anisocoria Nystagmus Ataxia Head pressing/holding	CL757	Q/Q
H887	54	2.4 x 10^6^	1.6 x 10^8^	Anisocoria Nystagmus Ataxia Head pressing/holding Partial hind limb paralysis	CL757	Q/Q
H842	90	5.2 x 10^6^	2.0 x 10^8^	None	CL757 and E543-3	TFP/Q
H843	95	2.1 x 10^5^	1.4 x 10^7^	None	CL757 and E543-3	TFP/Q

### Increased CSF Viral RNA is associated with NeuroAIDS

All animals showed peak CSF viral loads ranging from 10^2^ to 10^5^ viral RNA copies/mL between two and four-weeks post-infection (Fig 2B). These peaks were controlled down to the range of 10^3^ viral RNA copies/mL in the post-acute phases of infection. Viral loads in the CSF of four out of the eight animals infected (H880, H882, H886 and H887), ultimately reached the range of 10^7^−10^8^ viral RNA copies/mL (Fig 2B). Three of the four animals in this group (H880, H886 and H887) exhibited clinical symptoms of neuroAIDS (Table 1) that resulted in loss of motor control such ataxia, tremors and partial hind-limb paralysis, and other neurologic signs such as anisocoria, nystagmus, head pressing and hiding in the back of the cage. Onset of these symptoms required euthanasia according to animal care and use regulations [37]. All four of these animals exhibited brain lesions characteristic of SIVE/neuroAIDS (i.e., glial nodules, multinucleated giant cells and perivascular cuffing with macrophages and lymphocytes) by routine histopathology [38]. To confirm viral replication at the site of brain lesions, SIV-specific ISH was conducted to detect actively transcribed viral RNA. SIV RNA was detected at the site of brain lesions, indicating that brain lesions were associated with SIV replication (Fig 3B).

Despite robust replication, only four of the animals developed neuroAIDS. Some animals such as H881 and H885 developed opportunistic infections that required euthanasia. Since CSF viral load was on an upswing at the time of euthanasia, we speculated that these macaques may have eventually developed of CNS lesions if they had survived longer. Thus, although the CSF viral load of H881 increased in a similar manner to that observed in the four animals with SIVE, this animal developed a retro-orbital lymphoma at 48 weeks (most likely due to Rhesus Epstein Barr virus infection) while CSF viral load was in the early escalation phase. This necessitated euthanasia at 49 weeks post-infection. As for H885, the kinetics of increase in the CSF viral load was delayed to 32 weeks post-infection. CSF viral load of this animal reached to 10^4^ viral RNA copies/mL at 52 weeks post-infection and was euthanized at 53 weeks post-infection due to hind limb paresis but prior to the development of SIV encephalitis. The remaining two animals with lower set point plasma viral RNA loads, H884 and H883, also showed lower CSF viral load compared to the rest. However, these macaques showed increasing viral RNA levels in plasma and CSF at much later time-points than seen in animals with neuroAIDS (92 weeks post-infection for H883 and 44 weeks post-infection for H884). Although there was no obvious pathology in the CNS, CSF viral load of H883 and H884 reached 8 x 10^3^ and 5 x 10^4^ viral RNA copies/mL, respectively (Fig 2B) which is right at the threshold we previously observed to be associated with SIVE [39].

### Disease progression was associated with memory CD4+ T Cell depletion

Lymphocyte subset analysis revealed that CD4+ T cells decreased rapidly in the acute phase of infection (2 to 8 weeks post-infection) but showed transient recovery by 20 weeks post-infection, though overall the CD4+ T cells levels showed gradual decrease over time (Fig 2C). Loss of CD4+ T cells was more evident when we focused on CD4+ memory T cells. All animals were euthanized due to the onset of neurological and/or AIDS like symptoms (diarrhea, weight loss), which coincided with CD4+ T cell numbers of less than 200 cells/μL (Fig 2D).

### Compartmentalization of viral populations in CL757 infected animals

**Fig 4:**
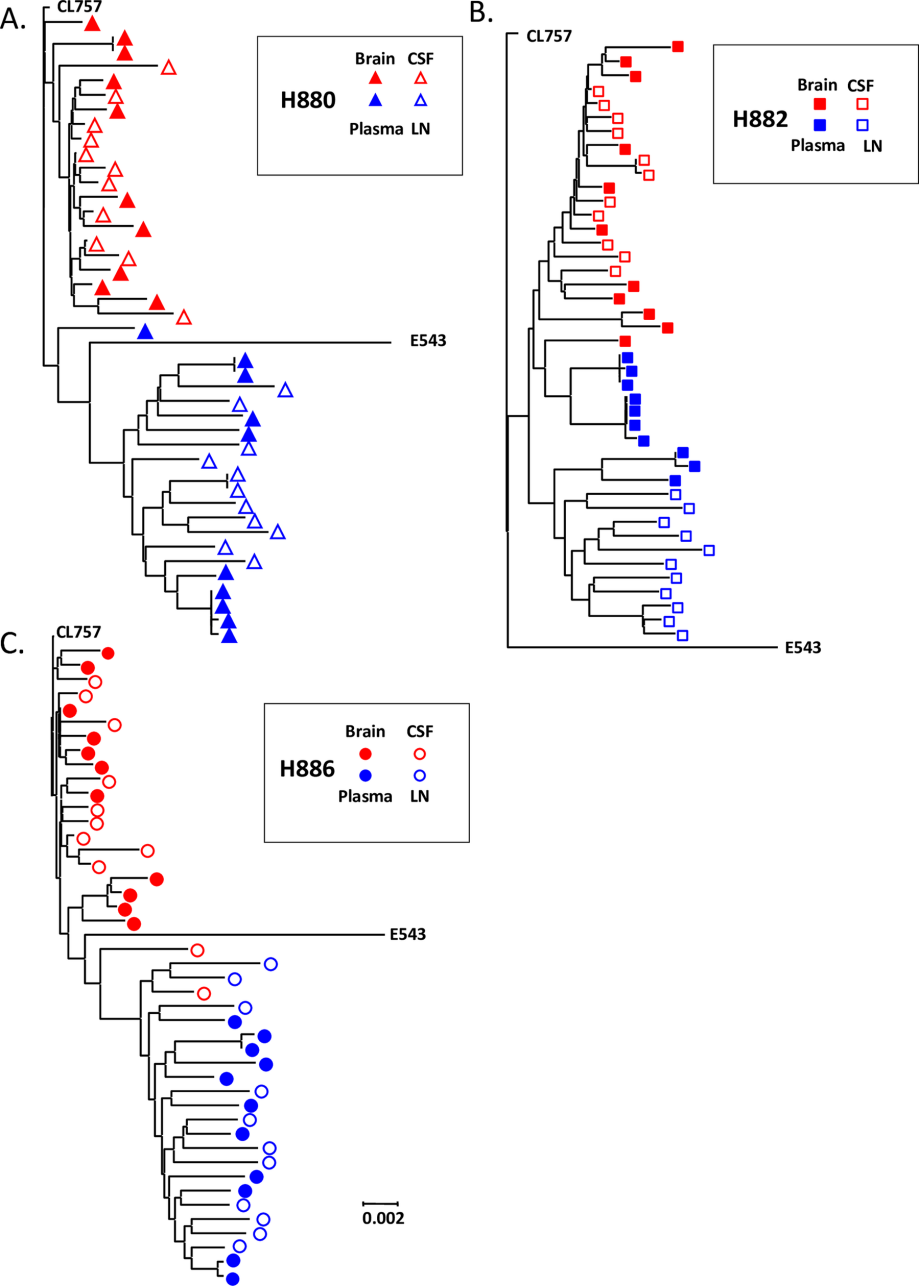
Compartmentalization of viruses in the CNS of animals inoculated with CL757 that developed SIVE. Phylogenetic analysis of envelope sequences amplified from the brain, CSF, plasma and axillary lymph node of animals (A) H880, (B) H882, (C) H886.

**Fig 5:**
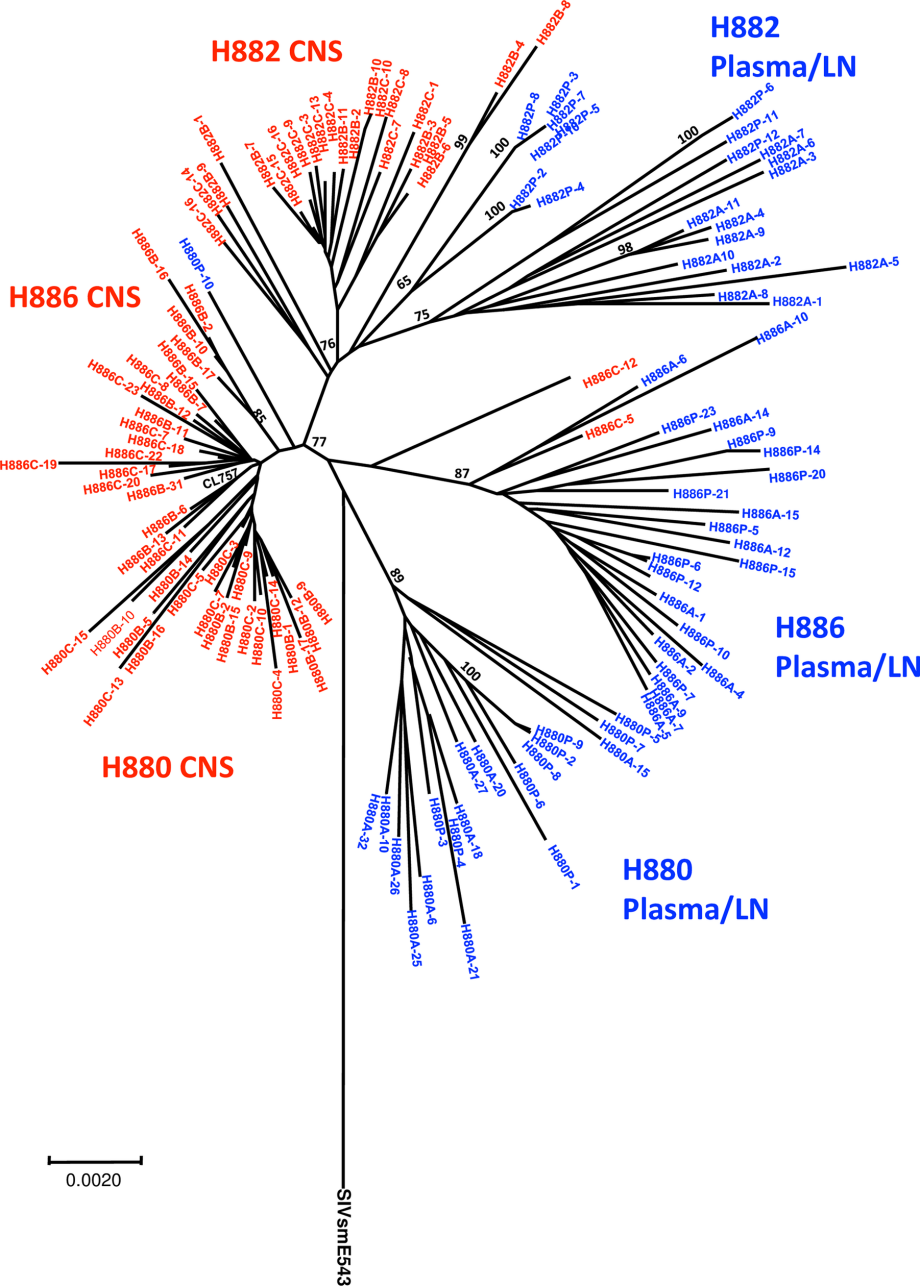
Phylogenetic analysis of envelope sequences amplified from the brain, CSF, plasma and axillary lymph node of animals infected with CL757 with sequences from all three animals combined in a radial tree demonstrates that viruses in the CSF/Brain from individual macaques were more closely related to one another than to the autologous plasma/lymph node sequences.

We and other groups have previously reported compartmentalization between plasma, CSF and brain parenchyma of animals infected with neurovirulent SIV or in patients that were infected with HIV-1 [27–32, 38]. However, these observations came from examination of viral populations in macaques inoculated with uncloned virus containing multiple variants or where the infecting strain was not identified such as in HIV-1-infected patients. Hence it was unclear whether compartmentalization was due to the acquisition of advantageous mutations by selective pressure, or whether the infecting virus contained variants that had advantages in replicating in certain compartments. To clarify which mechanism that drives compartmentalization in the CNS, full viral envelope fragments from the plasma, axillary lymph node, CSF and the brain parenchyma were amplified from samples collected at the time of necropsy from three animals that progressed to neuroAIDS (H880, H882 and H886). Phylogenetic analysis revealed clear compartmentalization between the periphery (plasma and axillary lymph node) and the CNS (CSF and brain parenchyma) in all of the animals (Fig 4A–4C). This finding suggests that interchange of viral populations between the CNS and the periphery are relatively rare in the end stage of the disease and viruses are likely to be produced locally within each compartment. To determine if the process of compartmentalization was similar in all three animals, we constructed a phylogenetic tree that included all variants from the three animals (Fig 5). Surprisingly, viruses from the CNS of all three animals formed a unique cluster distinct from the viral sequences obtained from the periphery, regardless of the animal of origin. Therefore, viruses from the CNS of these animals were more similar to one another than to variants in autologous plasma samples. Variants from the brain and CSF had relatively shorter branch lengths, indicating they are genetically closer to each other and the inoculum CL757 compared to those variants isolated from plasma and the lymph node. We analyzed consensus amino acid substitutions in each group to determine if the difference in branch length was due to higher level of viral replication in the plasma, or if there are selective pressures driving this divergence (Table 3). Although the substitutions were generally unique in each of the animals, we found some common mutations present within both the CNS and the periphery. As shown in Table 3, fewer common amino acid substitutions (9 positions) were observed in the CNS as compared to the periphery, consistent with the branch length in Fig 5. One amino acid substitution (S217G) was observed in H880 and H886. On the other hand, 31 amino acid substitutions were observed in variants isolated from the periphery. Thirteen of those amino acid substitutions were common in two or more animals, suggestive of common selective pressure for acquisition of those mutations in the periphery. Furthermore, there were five amino acids (D79N, A130T, N429K, A829T and R847K) that reverted to the residues found in E543-3, suggesting that these positions may be important for neurovirulence. Indeed, we have previously reported that amino acid substitution at T847A in 804E is associated with enhanced antagonism against host restriction factor BST-2 [23]. Reversion of this position to original E543-3 in periphery further supports idea that BST-2 restriction is important in the CNS microenvironment and SIVsm804E gained this mutation to counter act this restriction. In addition, reversion in this position observed in variants from plasma/LN compartments suggests that this mutation was negatively selected in these compartments.

**Table 3:** Summary of consensus substitutions in Env clones from the CNS and plasma/lymph node compartments of CL757-infected macaques.

		CSF/Brain			Plasma/Lymph Node		
Animal		H880	H882	H886	H880	H882	H886
**Region**	**Amino acid**						
**V2**	L4P				X		X
	D79N					X	
	A110T					X	X
	K115R						
	T116N					X	
	G125R						
	E126K				X	X	
	A130T					X	
	K134E						
	G152S						X
	K184T	X					
**V3**	#205M					X	
	Q212H						X
	S217G	X		X			X
	I338M					X	
**V4**	R348W		X			X	X
	A385T					X	X
	G386R					X	
	P389S					X	X
**V5**	N429K				X		X
	Q432R		X				
	Q433K					X	X
	Q435K		X				
	N455Y						X
	N519S					X	
	R520K		X			X	
**Gp41**	D634N				X		X
	F654S					X	
	G682N						X
	R768G				X		
	S793N				X		
	A829T				X		X
	R847K				X	X	
	I860M	X					
	R866G		X				
	G870R				X	X	X
	I874F					X	

### Co-inoculation of CL757 and SIVsmE543-3

**Fig 6:**
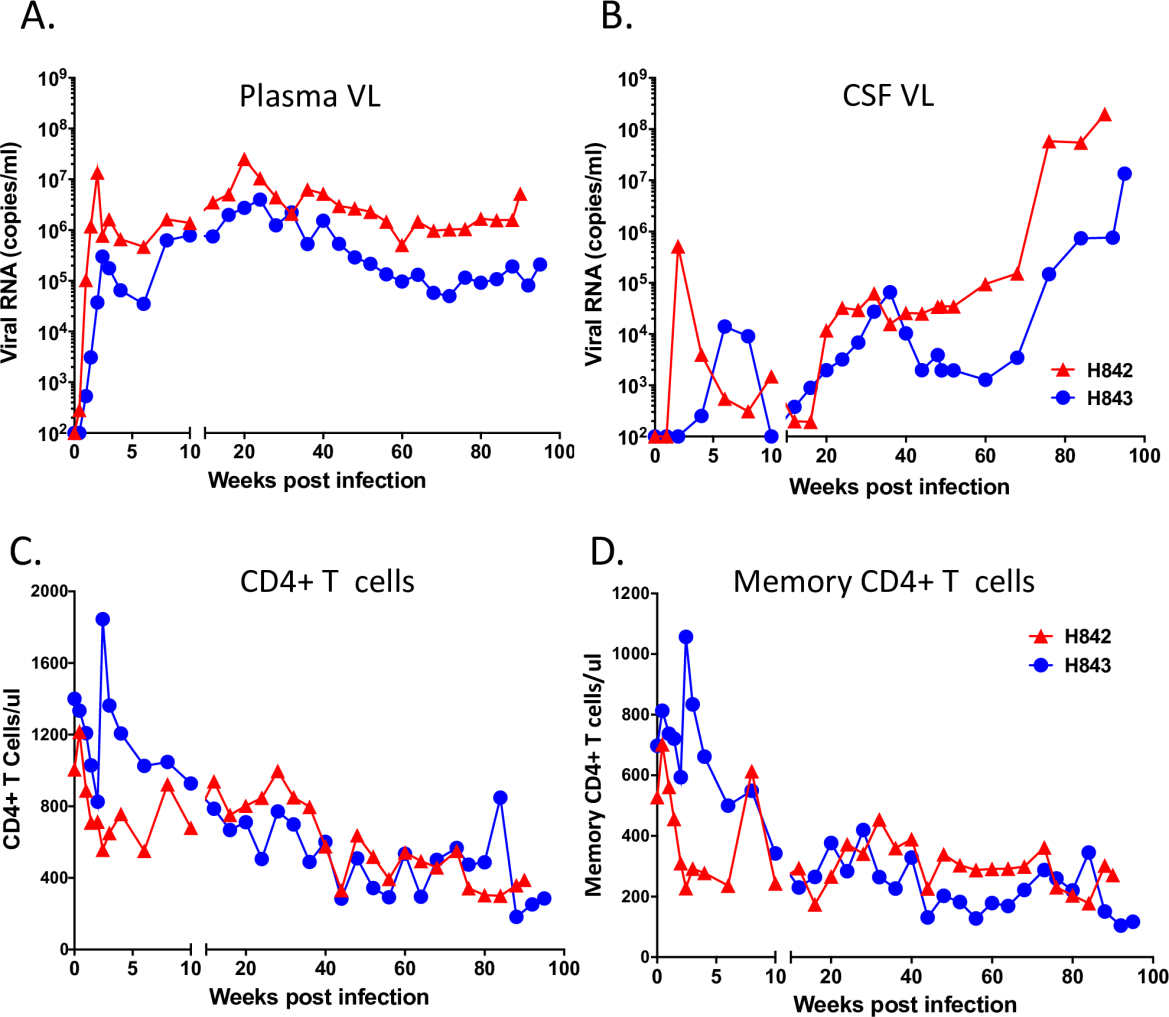
*In vivo* replication in animals co-inoculated with SIVsm804E-CL757 and SIVsmE543-3. (A) Plasma and (B) CSF viral RNA loads, and circulating (C) CD4+ T cells and (D) memory CD4+ T cells, of animals infected with SIVsm804E-CL757.

Phylogenetic analysis suggested that CL757 has advantage over its parent, E543-3 in the CNS microenvironment. To evaluate whether our neurovirulent CL757 specifically targeted the brain, we intravenously co-inoculated two animals with CL757 and E543-3 in a 1:1 ratio. Robust viral replication was observed in both infected animals with peak viremia of 1.4 x 10^7^ and 3 x 10^5^ viral RNA copies/mL at two weeks post-infection, respectively (Fig 6). This outcome is comparable with animals inoculated CL757 alone (Fig 2A). The set-point viral load of H842 and H843 were on par with animals that progressed to neuroAIDS (H880, H882, H886 and H887) ranging between 10^4^−10^7^ viral RNA copies/mL. The peak CSF viral load of H842, observed at 2 weeks post-infection (5.2 x 10^5^ viral RNA copies/mL) was highest among all the animals in this study. On the other hand, H843 showed delayed peak CSF viral RNA load at 6 weeks post-infection (1.4 x 10^4^ viral RNA copies/mL) (Fig 4B). CSF viral load for H842 and H843 declined to almost undetectable levels (200 viral RNA copies/mL at 12 weeks post-infection and undetectable level at 10 weeks post-infection, respectively). However, plasma viral load showed continuous increase throughout the course of disease. A significant increase in CSF viral load was observed at 76 weeks post-infection, exceeding plasma viral load in both animals (Fig 6A). Consistent with animals infected with CL757 alone, memory CD4+ T cells declined to approximately 200 cells/μL, for both animals by the time of euthanasia (Fig 6C). Viral RNA was detected at site of brain lesions in both H842 and H843, which is consistent with SIVE/neuroAIDS.

### Compartmentalization of SIV in co-inoculated animals

**Fig 7:**
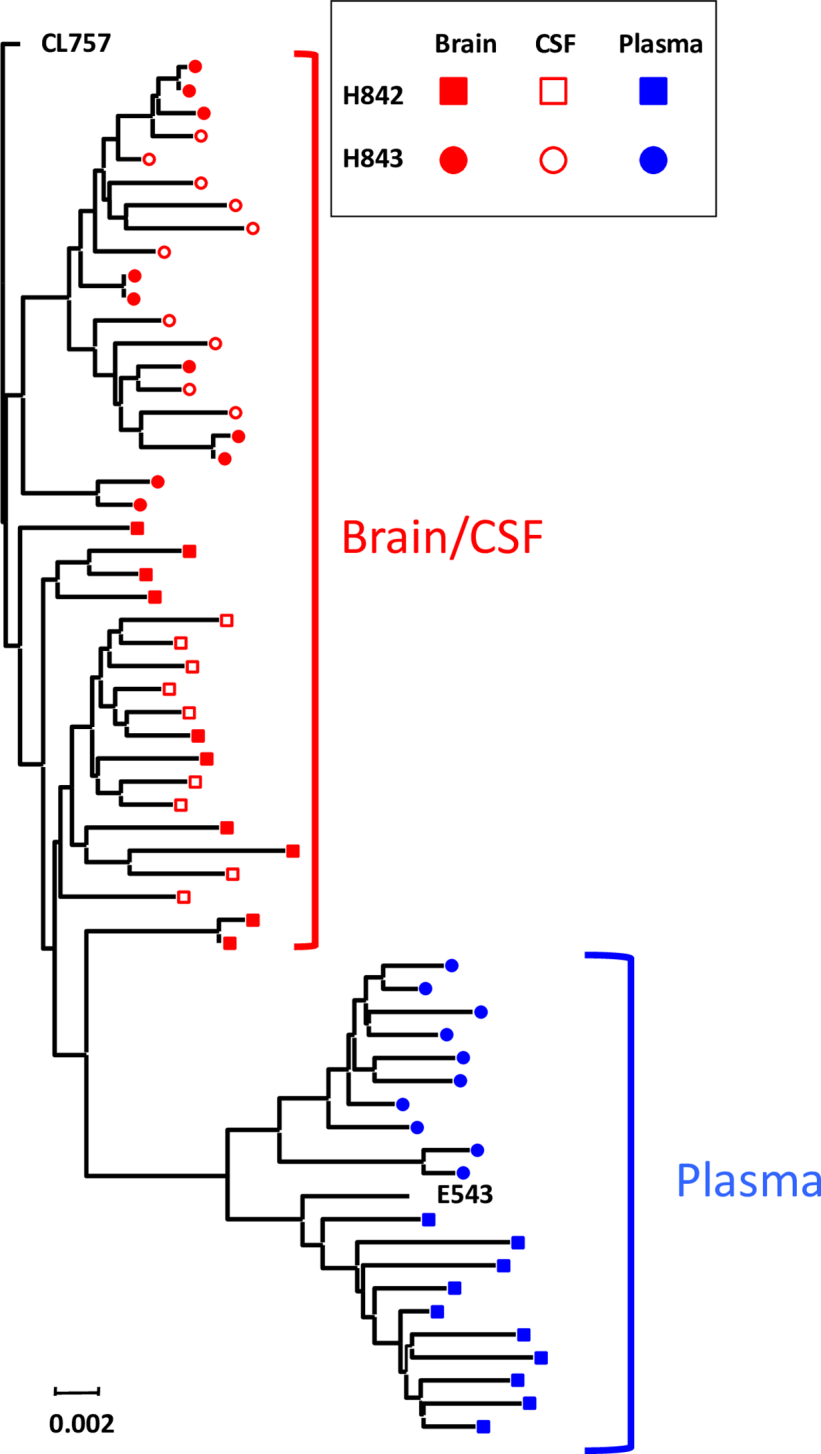
Phylogenetic analysis of envelope sequences amplified from the brain, CSF and plasma of animals co-inoculated with SIVsm804E-CL757 and SIVsmE543-3. Phylogenetic analysis of all envelope nucleotide sequences in H842 and H843 combined.

Previous studies using the pigtailed macaque model of co-inoculation of immunosuppressive SIVsmB670 with the neurovirulent 17EFr clone suggested that the clone specifically targeted the CNS but this finding was based on evaluation of a small region of env [40]. We conducted phylogenetic analysis of envelope sequences isolated from terminal plasma, CSF and the brain of H842 and H843 co-inoculated with CL757 and E543-3 (Fig 7). Consistent with results from animals inoculated with CL757 alone, compartmentalization of viral populations between the CNS and plasma was also observed in these two animals (Fig 7). More striking however was the compartmentalization of CL757-related variants, to the CNS, whereas variants from the plasma formed a group with E543-3, distinct from each other. This result clearly indicates that CL757 preferentially targets and replicates in the CNS whereas E543-3 has the advantage in replication in the periphery.

## Discussion

Shortly after infection, HIV becomes established in the CNS. In 20–30% of HIV-infected individuals it induces HIVE and the associated neurologic disorder HAD following a period of time that varies between individuals. Although the introduction of ART has reduced the incidence of the HAD, a milder form of neurologic disease, MCMD has become more common and is associated with a worse outcome for HIV patients. The virologic and biologic factors that determine whether an individual will develop neuroAIDS in any of its forms is not clear, nor is the extent to which the CNS represents a viral reservoir in ART-treated patients. Since neuropathological evaluation of HAD/MCMD progression is not feasible to study in humans due to the limited access to tissue samples, animal models that accurately recapitulate critical aspects of neuroAIDS in HIV-infected humans are necessary. In this study, we have developed a neuroAIDS model in rhesus macaques inoculated with either a neurovirulent virus, CL757 alone or a combination with its non-neurovirulent parent, E543-3. Unlike existing macaque models that produce a rapid onset of neuroAIDS, the disease progression in this current model closely resembles late stage development of neuroAIDS in HIV-infected patients.

These studies were initiated with the macaque passage of the molecularly cloned, AIDS-inducing SIVsmE543-3. Despite similarities in the nature of disease progression to that of HIV-1 infection in human, E543-3-infected rhesus macaques only rarely develop SIV encephalitis [22]. In an approach to establish a nonhuman primate model for neuroAIDS in rhesus macaques, we conducted sequential *in vivo* passage of E543-3, selecting viruses at each passage that had been isolated from the brain of animals that developed SIVE as the source of inoculum. Four such passages lead to the isolation of the uncloned virus SIVsm804E that induces neuroAIDS in a high proportion of rhesus macaques, if one excludes animals with restrictive TRIM5 α or MHC-I genotypes [22]. While this met many of the criteria we had established, disease progression in this model was variable with some animals developing a rapid disease course that is atypical of HIV neuroAIDS. Additionally the virus inoculum was quite complex leading to difficulty in molecularly tracking the virus(es) during disease progression. This made it a difficult model to dissect viral determinants of neurovirulence.

To further develop and improve this model for pathogenesis studies, we derived twelve full-length infectious viral clones from this uncloned 804E stock. Out of the 12 clones tested only one virus, CL757, was capable of replicating in both PBMCs and MDMs. Although CL757 showed a reduced capacity to replicate in PBMCs compared with its uncloned parental strain 804E, both showed similar replication kinetics and comparable levels of virus production in MDMs. Since replication of virus in macrophages in the CNS is a hallmark of neuroAIDS, CL757 was evaluated in rhesus macaques *in vivo*. Robust viral replication was observed in the eight inoculated animals as evidenced by high acute plasma viral RNA levels, similar to levels as we previously reported with uncloned 804E [22] and virus was detectable in the CSF of each of the animals during primary infection and reached high levels in 50% of the animals following a variable eclipse phase. Importantly, this increase was associated with development of SIVE/neuroAIDS. Most of the animals infected showed high levels of viral RNA in the CSF at the time of necropsy, although this was particularly evident in the animals that progressed to SIVE (H880, H882, H886 and H887). Three of the four (H880, H886 and H887) presented with neurological symptoms that necessitated euthanasia. These clinical symptoms of neurological disease included behavioral issues and loss of motor control, similar to symptoms in HAD/MCMD [41, 42]. Each of these animals also met the clinical definition of AIDS as assessed by depletion of CD4+ T cells below approximately 200 cells/μl, indicating that immune suppression may be essential for the development of neuroAIDS. Post-mortem examination of brain tissue of all four animals with neuroAIDS revealed widespread lesions in the white matter characterized by perivascular cuffing and glial nodules containing multinucleated giant cells expressing SIV RNA as measured by ISH [43]. We did not observe rapid disease progression in any of the animals infected with CL757 or the two animals co-inoculated with E543-3 and CL757. The more conventional disease progression contrasted with the more rapid disease observed in animals inoculated with uncloned SIVsm804E [22]. The lack of rapid disease is fortunate in that it will provide an excellent model to assess mechanisms of disease progression in the CNS in the future, and is likely more reflective of the late stage disease progression in HIV-1 infected individuals.

Due to the use of molecularly cloned virus, this study also allowed us to examine the molecular characteristics and evolution of virus in the peripheral immune system and the CNS. Previous studies in both HIV-infected humans and SIV-infected macaques show that the microenvironment of the CNS fosters viral compartmentalization [27–32, 38]. However, it is not clear if viruses become compartmentalized in the CNS due to a founder effect where specific viruses target the brain or whether compartmentalization occurs through the selection and evolution in this compartment. We previously reported viral compartmentalization in animals inoculated with uncloned virus stocks (SIVsm783Br and SIVsmH445) where the inoculum is complex and individual components are difficult to track [38, 44]. In the present study, the analysis of viral compartmentalization in macaques inoculated with CL757 allowed us to better comprehend the evolution and selective events involved in development of neuroAIDS. Additionally, using co-inoculation of two molecular clones, one neurovirulent (CL757) and the other AIDS-inducing but rarely neurovirulent (E543-3) we were able to confirm that CL757 is clearly highly adapted for replication in the CNS in contrast to its parent E543-3 strain which was generally excluded from the CNS. Phylogenetic analysis of CL757-infected animals revealed that variants isolated from CSF and the brain formed a clade distinct from variants isolated from plasma and lymph node. This result indicates distinct selective pressures between the CNS and the periphery. The CNS group had a lesser degree of divergence than that of plasma/lymph node group, suggesting that there are selective pressures against replication of CL757 in plasma/lymph node. Alternatively, greater and more sustained levels of viral replication in the plasma/lymph node compartment allowed virus to accumulate mutations. Upon closer examination, we saw mutations in the envelope of variants isolated from the periphery that appear in the parental clone E543-3 (D79N, A130T, N429K, A829T and R847K). These mutations were not observed in the variants isolated from the CNS suggesting that CL757 is highly adapted to replicate in the CNS, whereas E543-3 has advantage in the periphery. Co-inoculation of both E543-3 and CL757 confirmed this hypothesis. The variants from CNS formed one group with CL757 sequence, whereas variants from plasma and lymph node formed another group with E543-3. Thus, those amino acids that reverted back to original E543-3 may play important role in neurovirulence, which may have disadvantages for replication in plasma and lymph node. Indeed, we have previously reported that amino acid substitution at position T829A in E543-3 enhances antagonism against the host restriction factor BST-2 [23]. The fact that this amino acid substitution reverted back to the original E543-3 residue (A829T) in the plasma and lymph node indicates that enhanced BST-2 antagonism is positively selected in the CNS but negatively selected in the plasma and lymph node. The evolution of CL757 was associated with a change in biological properties. The most obvious change was the acquisition of the ability to replicate efficiently in MDMs, which has implications for neurovirulence. Our prior study demonstrated that differences in the cytoplasmic tail of gp41 (gp41 CT) of the neurovirulent variant were associated with enhanced replication in MDMs and better antagonism of BST-2 [23]. While introduction of these substitutions to E543-3 enhanced replication in MDMs and virus levels in the CSF, they did not fully recapitulate the neurovirulence of CL757, indicating that other differences are important for neurovirulence.

In summary, we have successfully isolated a molecular clone of SIV that is highly adapted to target and replicate efficiently in the CNS microenvironment and induce neurological disorders in infected rhesus macaques in high frequencies. The disease course of SIVsm804E-CL757 infected animals is conventional as opposed to those existing rapid disease models that require immunomodulation or that require the use of pigtail macaques. The CL757 model will enable us to assess mechanisms of disease progression in the CNS during the chronic phase of infection. CL757 shares the same genetic backbone with its parental E543-3, therefore this virus would be a powerful tool to assess viral determinants of neurovirulence.

## Materials and methods

### Ethics statement

This study was carried out in strict accordance with the recommendations described in the Guide for the Care and Use of Laboratory Animals of the National Institute of Health, the Office of Animal Welfare and the United States Department of Agriculture. Colony-bred rhesus macaques of Indian origin were obtained from Morgan Island rhesus monkey breeding colony, SC. All animal work was approved by the NIAID Division of Intramural Research Animal Care and Use Committees (IACUC), in Bethesda, MD (Animal Study protocol, #ASP-LMM-6). The animal facility is accredited by the American Association for Accreditation of Laboratory Animal Care. All procedures were carried out under Ketamine anesthesia by trained personnel under the supervision of veterinary staff and all efforts were made to ameliorate the welfare and to minimize animal suffering in accordance with the “Weatherall report for the use of non-human primates” recommendations. Animals were housed in adjoining individual primate cages allowing social interactions, under controlled conditions of humidity, temperature and light (12-hour light/12-hour dark cycles). Food and water were available ad libitum. Animals were monitored twice daily (pre- and post-challenge) and fed commercial monkey chow, treats and fruit twice daily by trained personnel. Early endpoint criteria, as specified by the IACUC approved score parameters, were used to determine when animals should be humanely euthanized.

#### Construction of a full length molecular clone SIVsm804E-CL757

Construction of SIVsm804E-CL7E7 was described previously [23]. Briefly, viral RNA was isolated from the SIVsm804E viral stock by QIAamp Viral RNA Mini Kit (QIAGEN, Venlo, Netherlands). The viral RNA was then reverse transcribed by ThermoScript RT-PCR System (Thermo Fisher Scientific, Waltham, MA) using gag-R or R-R reverse primers, which covers 5’ and 3’ LTR sequences [23]. PCR was performed on each resultant cDNA by Platinum *taq* Hi Fidelity Kit (Thermo Fisher Scientific, Waltham, MA) using R-F and gag-R, or Bgl-F and R-R, respectively. Two PCR products were then mixed at a 1:1 ratio and incubated at 95°C for 5 minutes then cooled down to the room temperature to allow annealing of the two products at the shared R region. A complete LTR with partial *env* sequence on the 5’ side and primer binding site on the 3’ side was obtained by overlapping PCR of above product by Platinum *taq* Hi Fidelity Kit using Bgl-F and gag-R as primers. This product was then TA cloned into the pCR4-TOPO vector using a TA cloning kit (Thermo Fisher Scientific, Waltham, MA), resulting in 1LTR TOPO, and sequenced. To create 2LTR puc19 with unique restriction sites that allow rest of the coding regions to be transferred, 1LTR TOPO clones were digested with NdeI and NarI restriction enzymes and transferred into puc19 vector (New England Biolabs, Ipswich, MA), resulting in 1LTR puc19. 1LTR TOPO vector was digested with EcoRI and NarI and the fragment with LTR sequence was introduced into the region between EcoRI and SmaI of 1LTR puc19 vector to create a 2LTR puc19 vector. The wild type SIVsmE543-3 full-length plasmid was digested with NarI and BglII restriction enzymes and inserted into the region between the same restrictions sites of 2LTR puc19 vector to obtain three full-length vectors, CL4E4, CL6E6 and CL7E7. To obtain full length clones of the 804 virus stock, PBMCs were isolated from whole blood of SIV naïve, healthy rhesus macaques, stimulated with 5ug of phytohemagglutinin (PHA) per ml and 10% interleukin-2 (IL-2) (Advanced Biotechnologies, Columbia, MD) for 72 hours, and maintained in RPMI 1640 medium containing 10% fetal calf serum (FCS) and 10% IL-2. PBMCs (5 x 10^5^ cells) were inoculated with SIVsm804E at a MOI of 0.01 and incubated for 3 days. Cells were then collected and viral DNA was extracted with QIAamp DNA Blood Mini Kit (Qiagen, Santa Clara, CA). PCR was performed with Herculase II Fusion DNA polymerase (Agilent Technologies, Hercules, CA) using primers NarI-F (5’- GGT TGG CGC CCG AAC AGG GAC GAC TT -3’) and NdeI-R (5’- CAG AGT CTC CCA TAT GTC TCT CC -3’). Amplified viral DNA fragment was cloned into pCR Blunt II-TOPO vector using Zero Blunt TOPO cloning Kit (Thermo Fisher Scientific). The plasmid containing the insert was then digested with NarI and Bgl II restriction enzymes and the insert was ligated into the corresponding regions of CL4E4, CL6E6 and CL7E7 [23] to obtain twelve unique chimeras including CL757.

#### Viral stocks

CL757, E543-3 and SIVmac239 viral stocks were obtained by transfection of 293T cells (AIDS reagent program, catalog 103) with full-length molecular clones using Fugene 6 Transfection Reagent (Promega). Supernatants were collected 48 hours after transfection and cryopreserved in -80°C until use. SIVsm804E viral stock was prepared as described previously [22]. The 50% tissue culture infectious dose (TCID50) of all virus stocks was measured by the TZM-bl assay as described elsewhere [45].

#### Viral replication in PBMCs and MDMs

Virus replication of the CL757 was evaluated in rhesus macaque PBMCs and MDMs. Rhesus macaque MDMs were purified from freshly isolated PBMCs by incubation with anti-nonhuman primate CD14 magnetic beads (Miltenyi Biotec, Auburn, CA) followed by positive selection with magnetically activated cell sorting (MACS) separation columns (Miltenyi Biotec). A total of 3 x 10^5^ cells per well of CD14^+^ cells were cultured in a 48-well plate for 4 days in RPMI 1640 medium containing 10% FCS, 10% human serum type AB (Sigma, St. Louis, MO), and 20 ng/ml of macrophage colony-stimulating factor (R&D systems, Minneapolis, MN). Wells were washed two times with Hanks Balanced salt solution (HBSS) and cultured in fresh medium for three additional days. PBMCs (5 x 10^5^ cells/ well) were dispensed into 48-well plate and then inoculated with each virus at MOI of 0.01. MDMs were incubated with virus at an MOI of 0.01 for 1 hour and then washed twice with HBSS and cultured in fresh medium. Virion-associated RT activity of the culture supernatant was monitored periodically [46].

#### *In vivo* replication

Naïve Indian origin rhesus macaques (SIV, simian retrovirus [SRV], and simian T cell leukemia virus type 1 [STLV-1] seronegative) were prescreened for MHC-I and TRIM5 genotype [47, 48]. None of animals used in this study expressed MHC-I genotypes that are known to be restrictive to SIVmac239 (i.e., A1*001, B*008 B*017, B*029) [49–53]. As shown in Table 1, five animals expressed a moderately susceptible TRIM5 genotype (H880 and H881 with TFP/Q; H882, H883, and H884 with Q/Cyp) and 3 animals with highly susceptible TRIM5α genotypes (H885, H886 and H887 with Q/Q) were inoculated intravenously (i.v.) with 500 TCID50 of CL757. Two additional animals with a moderately susceptible TRIM 5 genotype (H842 and H843 with TFP/Q) were co-inoculated intravenously with 500 TCID50 of CL757 and 500 TCID50 of E543-3 (Table 1). Plasma and CSF viral RNA loads were monitored periodically. All animals were housed in accordance with American Association for Accreditation of Laboratory Animal Care standards. The investigators adhered to the *Guide for the Care and Use of Laboratory Animals* [37] and to NIAID Animal Care and Use Committee-approved protocols.

#### Detection of viral RNA expression in the CNS

Formalin-fixed, paraffin embedded brain sections were assayed for SIV RNA expression by *in situ* hybridization (ISH) as previously described [43]. Briefly the sections were hybridized overnight at 45°C with either a sense or an antisense digoxigenin-UTP-labeled SIVmac239 riboprobe that spanned the entire genome. The hybridized sections were blocked with 3% normal sheep and horse sera in 0.1M Tris, pH 7.4, and then incubated with sheep anti-digoxigenin-alkaline phosphatase (Roche Molecular Biochemicals) and nitroblue tetrazolium-5-bromo-4-chloro-3-indolyl-β-D-galactopyranoside (BCIP; Vector Laboratories), followed by counterstaining with Nuclear Fast Red solution (Sigma). ISH-stained tissues were visualized and photographed with Zeiss Axio Imager Z1 microscope (Zeiss)

#### Flow cytometric analysis

Absolute CD4+ T cell or memory CD4+ T cell counts in the blood were monitored using a BD-LSR Fortessa with DiVa software (v6.0). PBMCs were stained with antibodies to CD3 (557917; BD Pharmingen), CD8 (558207; BD Pharmingen), CD4 (35004873; eBioscience), CD95 (559773; BD Pharmingen), CD28 (6607111; Beckman Coulter) and an Aqua Live/Dead stain (L34957; Invitrogen) then washed with phosphate-buffered saline (PBS), followed by fixation with 0.5% paraformaldehyde and analyzed using a Fortessa fluorescence-activated cell sorter (FACS). Absolute memory CD4+ T cells were determined using CD28 and CD95 as markers. Data analysis was performed using Flowjo (v9.3; TreeStar, San Carlos, CA).

#### Neutralization assay

SIV-specific neautralizing antibodies (Nabs) were evaluated using the TZM-bl neutralization assay as previously described [36]. Briefly, serial diluted heat-inactivated serum samples were mixed with 50 TCID_50_ viruses and incubated at 37°C for 1 hour. After incubation, 10^4^ TZM-bl cells were added to each well with DEAE-dextran at a final concentration of 12.5 μg /ml to enhance virus infectivity. After 40 hours of culture at 5% CO2 and 37°C, luciferase activity was measured with the Luciferase assay kit (Promega) and read on Mithras LB940 (Berthold technologies). Average relative luminescence units (RLU) of cell controls were subtracted as background and the percent inhibition plotted for each serum dilution.

#### Viral RNA extraction and sequence analysis

CSF, plasma, brain and the axillary lymph nodes were collected from animals at the time of necropsy and cryopreserved. The viral RNA was extracted using a QIAamp Viral RNA Mini Kit (Qiagen). All animals used in this study were perfused with saline prior to sample collection to avoid blood contamination in the tissues. Fresh brain tissue was collected from the frontal, parietal, and temporal lobes, the cerebellum, and the midbrain during necropsy and homogenized together using the cell strainer to obtain single cell suspensions. Fresh axillary lymph node was collected and homogenized using the cell strainer to obtain single cell suspension. Total RNA was extracted from the brain and axillary lymph node using RNeasy Mini Kit (Qiagen) and then treated with DNase I (Invitrogen, Grand Island, NY) to digest residual genomic DNA. The viral RNA was reverse transcribed using a Thermoscript reverse transcriptase PCR (RT-PCR) system (Invitrogen) and the primer 9341-R (CATCATCCACATCATCCATG). The envelope DNA fragment was amplified by PCR with Platinum taq DNA polymerase (Invitrogen) and primers 6463-F (GGTGTTGCTATCATTGTCAGC) and 9341-R and then subcloned into the pCR4-TOPO vector by use of a TOPO TA cloning kit (Invitrogen) for sequencing. A minimum of 10 clones were selected for each sample from each of five macaques, and the complete envelope gene was sequenced.

#### Phylogenetic and sequence analysis

Full length SIV envelope sequences were aligned using the Clustal W program from the MacVector 15.1.1 software suite (MacVector Inc, Apex NC USA) with minor manual adjustments. Phylogenetic trees were constructed using the neighbor-joining method using Mega 6.06 software suite. The evolutionary distances were estimated using the maximum composite likelihood method and are expressed as number of substitutions per site. Bootstrap values were determined using 500 replicates. Consensus sequences of Env clones from each of three macaques inoculated with CL757 sampled from CSF/Brain and Plasma/Lymph node were defined as the nucleotides present in the majority of clones (50%) from an animal and compartment. Sequences have been deposited in GenBank as follows:

Full length CL757 clone MF37082;
SIVsm804E stock sequences MF357903 to MF357922;
H880 sequences = MF370654 to MF370695;
H882 sequences to MF370696 to MF370738;
H886 sequences = MF370739 to MF370782;
H842 sequences = MF370783 to MF370811;
H843 sequences = MF370812 to MF370841
